# Ethnic Identity as a Driver of Supportive Consumer Decisions: A Behavioral Perspective

**DOI:** 10.3390/bs16020225

**Published:** 2026-02-03

**Authors:** Matti Rachamim, Ori Grossman, Yossi Mann

**Affiliations:** 1 Graduate School of Business Administration, Bar-Ilan University, Ramat Gan 5290002, Israel; ori-avraham.grossman@biu.ac.il; 2 The Department for Middle Eastern Studies, Bar-Ilan University, Ramat Gan 5290002, Israel; yosef.man@biu.ac.il

**Keywords:** ethnic identity, consumer behavior, minority-owned businesses, product involvement, social identification, loyalty intentions, store evaluations

## Abstract

Ethnic identity is a central psychological construct that shapes social perception, intergroup behavior, and everyday decision making, yet its influence on support for minority-owned businesses remains insufficiently understood. This research examines how variations in ethnic identification predict consumer preferences, evaluations, and loyalty, and whether these effects depend on product involvement. Two empirical studies were conducted among members of an ethnocultural minority group. Study 1 assessed preferences for minority versus majority-owned businesses across four purchase scenarios differing in involvement level. The findings show that ethnic identity predicts supportive choices primarily in low-involvement contexts: individuals with stronger ethnic identification were more likely to prefer minority-owned supermarkets, and indicators of cultural attachment predicted preference for minority-owned restaurants. No identity effects emerged in high involvement decisions, where utilitarian considerations dominated. Study 2 examined whether ethnic identity predicts store evaluations and loyalty toward a minority-owned retail chain. Across both involvement conditions, stronger ethnic identification was associated with more positive store evaluations, greater perceived value, higher fairness assessments, and stronger loyalty intentions, while product involvement and its interaction with identity were nonsignificant. Together, the results demonstrate that ethnic identification meaningfully shapes consumer support for minority enterprises and clarify the conditions under which identity-based processes guide marketplace behavior.

## 1. Introduction

Entrepreneurship among ethnic minority groups constitutes an important pathway for socioeconomic participation, mobility, and community resilience. Although minority-owned ventures have become increasingly visible, they continue to encounter substantial structural constraints, including limited access to capital, weaker business networks, and reduced exposure to mainstream consumer markets (Aldrich & Waldinger, 1990; Fairlie & Robb, 2008). These barriers contribute to persistent disparities in entrepreneurial outcomes and have prompted scholars to reconsider whether minority status influences business performance through mechanisms that extend beyond financial or institutional disadvantage. Traditional explanatory models, such as the Lussier (1995) success–failure framework, emphasize managerial, financial, and environmental determinants of business viability, while treating minority status primarily as a demographic attribute with limited behavioral interpretation (Gyimah et al., 2019).

Empirical evidence based on these models remains mixed. Some studies report higher failure rates among minority-owned ventures, attributing these outcomes to structural inequalities in credit access and business opportunities (Cooper et al., 1990; Bennett & Robinson, 2024). Other research finds no systematic differences once managerial competence and planning are controlled for (Lussier & Pfeifer, 2001; Staber & Aldrich, 1995). These inconsistencies indicate that minority entrepreneurship may be shaped not only by material constraints but also by psychological and social processes that influence how businesses are perceived, evaluated, and supported in the marketplace.

Contemporary perspectives from marketing and social psychology highlight the centrality of ethnic identity as a determinant of consumption patterns and marketplace judgments. Ethnic identity, a sense of belonging, emotional attachment, and cultural commitment to one’s group (Phinney, 1992; Laroche et al., 2007; Cleveland et al., 2015) serve as a motivational force that shapes how individuals evaluate products, services, and businesses. Consumers frequently draw on identity-related cues to express belonging and maintain social ties (Belk, 1988; Reed, 2002; Oyserman, 2009). Recent work also shows that consumers from minority backgrounds may rely on identity-based reasoning in forming perceptions of fairness, trust, and social alignment in marketplace interactions (Oyserman, 2009). Under such conditions, supporting minority-owned enterprises becomes not only an economic act but also a symbolic expression of group solidarity and collective continuity. Research on identity signaling and identity-based motivation further demonstrates that salient group membership can influence attitudes, fairness judgments, and loyalty toward in-group actors (Crocker & Luhtanen, 1992; Berger & Heath, 2007; Reed et al., 2012; Reed et al., 2007).

Identity-driven behavior is conditioned by the cognitive and emotional demands of the decision context. The consumer involvement literature shows that low-involvement purchases are guided more heavily by affective and symbolic cues, whereas high-involvement decisions require greater deliberation and emphasize utilitarian considerations such as price, reliability, and perceived risk (Laurent & Kapferer, 1985; Celsi & Olson, 1988; Ahmed et al., 2004). Recent behavioral findings further demonstrate that reliance on identity cues decreases when cognitive load or task complexity increases (Forehand et al., 2011). Research grounded in dual-process models similarly suggests that identity-based effects become less potent when cognitive elaboration is high or when decisions carry greater financial or functional stakes (Petty & Cacioppo, 1986; Bettman, 1979; Zaichkowsky, 1985; Cleveland et al., 2015). These findings emphasize that the impact of ethnic identity depends not only on the strength of identification but also on the complexity and stakes of the decision environment.

Understanding how ethnic identity operates across varying levels of involvement is essential for interpreting consumer support for minority-owned businesses in multicultural societies. Identity-driven consumption can reinforce social cohesion, strengthen local economic ecosystems, and mitigate structural disadvantages faced by minority entrepreneurs (Dana, 2011; Essers & Benschop, 2009). Empirical work on ethnic consumption further illustrates that group-based affinity can translate into sustained support for minority-owned enterprises, particularly in symbolic or community-oriented domains (Karoui et al., 2023). Conversely, when identity alignment is limited, minority ventures may confront both market exclusion and insufficient in-group support.

The present research draws on social identity theory (Tajfel & Turner, 1979), identity-based motivation (Oyserman, 2009), and consumer involvement frameworks (Laurent & Kapferer, 1985; Zaichkowsky, 1985) to investigate how ethnic identification predicts supportive consumer behavior toward minority-owned businesses. Two complementary studies assess whether ethnic identity influences: (a) preferences between minority- and majority-owned businesses across different purchase contexts, and (b) evaluations and loyalty intentions toward a minority-owned retail chain.

By integrating psychological and contextual perspectives, this work expands the understanding of minority entrepreneurship by positioning consumers as active agents whose identity-related motivations shape market interactions and, ultimately, the sustainability of minority enterprises.

## 2. Literature Review

### 2.1. Minority Entrepreneurship and Business Outcomes

Minority entrepreneurship has long been recognized as a key driver of socioeconomic mobility, innovation, and local development, yet the outcomes of such ventures often diverge from those of majority-owned businesses (Aldrich & Waldinger, 1990; Dana, 2011). Structural constraints, limited access to financial capital, and social barriers have been found to reduce the survival rates of minority enterprises, contributing to persistent disparities in business performance (Fairlie & Robb, 2008). Within this context, minority status has typically been treated as an exogenous risk factor, reflecting constraints in credit markets, business networks, and institutional support.

One of the most established frameworks for predicting entrepreneurial success or failure, the Lussier model (Lussier, 1995), emphasizes the combined influence of entrepreneur characteristics, business characteristics, and macroeconomic conditions. Minority status has often appeared in this framework as a background variable, signaling potential vulnerability due to structural disadvantage rather than as a psychologically meaningful construct in its own right (Gyimah et al., 2019). Empirical findings derived from this and related approaches remain inconclusive. Some studies report higher failure rates among minority entrepreneurs and attribute these outcomes to inequalities in access to finance and opportunities for growth (Cooper et al., 1990; Fairlie & Robb, 2008). Other work finds no systematic differences once factors such as managerial competence, education, and industry are taken into account (Lussier & Pfeifer, 2001; Staber & Aldrich, 1995). Taken together, these inconsistencies suggest that minority entrepreneurship cannot be understood solely through financial or structural variables.

An emerging line of research suggests that the interaction between social identity, consumer behavior, and cultural context plays a meaningful role in shaping entrepreneurial outcomes. Minority entrepreneurs operating within communities that share their ethnic background may benefit from symbolic solidarity, trust, and consumer support that partially compensate for structural disadvantages. This perspective underscores the importance of examining demand-side mechanisms, and specifically how ethnic identity translates into marketplace preferences and loyalty patterns that sustain minority ventures and ethnic enterprises embedded within their communities.

### 2.2. Ethnic Identity and Consumer Behavior

Ethnic identity refers to the degree to which individuals identify with, feel attached to, and derive emotional significance from their ethnic group membership (Phinney, 1992). It encompasses both cognitive elements of self-categorization and affective components such as pride and belonging (Crocker & Luhtanen, 1992). In the consumer domain, ethnic identity has been shown to shape brand evaluation, product preference, and store choice, particularly when cultural cues or ownership signal group affiliation (Laroche et al., 2007; Cleveland et al., 2015). In the present research, ethnic identity is conceptualized as identification with one’s own ethnic group, rather than as generalized identification or solidarity with minority groups more broadly.

The concept of consumer ethnocentrism further clarifies this link. Consumer ethnocentrism describes a moral and affective predisposition to favor products and businesses associated with the in-group and to resist out-group alternatives perceived as threatening local welfare (Sharma et al., 1994). Relatedly, in-group favoritism as articulated in social identity research (Tajfel & Turner, 1979) provides a social-psychological foundation for understanding why consumers may prefer to patronize businesses owned by individuals who share their cultural or national identity. Such consumption patterns serve not only economic but also symbolic functions, reinforcing social cohesion and affirming collective self-esteem (Crocker & Luhtanen, 1992).

At the same time, the influence of ethnic identity on consumer behavior is not uniform. Contextual factors such as product type, social setting, and level of involvement have been shown to moderate the strength of identity-based preferences (Ahmed et al., 2004). For example, consumers may express ethnic solidarity more strongly in daily, low-involvement purchases, such as groceries or dining out, where decisions are guided more by emotional and social motives than in high-involvement or risk-intensive transactions that require extensive deliberation. Processes of globalization and acculturation further complicate these patterns, as many consumers negotiate tensions between cultural loyalty and cosmopolitan openness, resulting in ambivalent attitudes toward ethnic versus mainstream businesses (Josiassen, 2011; Shoham et al., 2017). In this broader perspective, supporting minority-owned businesses can function simultaneously as an economic choice and as a symbolic act of in-group solidarity.

### 2.3. Self-Concept, Social Identity, and Market Behavior

The impact of ethnic identity on consumer decisions can be situated within broader theories of self-concept and social identity. Self-concept theory posits that individuals construct and maintain their sense of self through possessions, affiliations, and symbolic forms of consumption (Belk, 1988; Reed, 2002). Products, brands, and retail environments become extensions of personal and collective identity, enabling consumers to express who they are and which groups they belong to (Aaker, 1999). From this viewpoint, marketplace choices are not merely responses to functional attributes but also tools for managing and communicating identity.

Social identity theory (Tajfel & Turner, 1979) complements this perspective by emphasizing that people derive part of their self-esteem from membership in social groups. Favoring in-group members, including entrepreneurs or brands representing the in-group, can enhance perceived social standing and strengthen intergroup distinctiveness. When ethnic identity is salient, consumers tend to evaluate in-group products and services more positively, sometimes even when objective quality differences are small or ambiguous (Cleveland et al., 2015). This dynamic is closely related to identity signaling, whereby consumers use marketplace choices to communicate belonging, values, and social alignment to others (Berger & Heath, 2007).

However, identity-based consumption is also sensitive to the broader sociopolitical context. In polarized or conflictual environments, identity expression through consumption can carry reputational or moral implications (Russell & Russell, 2006). In such settings, supporting in-group businesses may function both as a form of economic participation and as a subtle expression of social resistance or affirmation. Hence, the same act of patronizing a minority-owned business may be interpreted as a routine purchase in one context or as a meaningful statement of solidarity in another, depending on prevailing intergroup relations.

### 2.4. Product Involvement and Identity-Sensitive Consumption

Product involvement reflects the perceived personal relevance, risk, and emotional engagement associated with a purchase (Laurent & Kapferer, 1985; Zaichkowsky, 1985). It influences how much cognitive effort consumers invest in information processing and decision-making (Celsi & Olson, 1988). Low-involvement purchases, such as everyday groceries or casual restaurant visits, tend to rely more on habits, affective reactions, and identity-related cues, whereas high-involvement decisions, such as buying technology or choosing financial services, invite more analytical evaluation (Ahmed et al., 2004).

From an identity perspective, involvement level moderates the salience and impact of social motives. When cognitive processing demands are relatively low, individuals are more likely to rely on heuristics, including in-group affiliation and cultural compatibility, as guides to choice (Petty et al., 1983). Under these conditions, ownership cues and perceived shared identity can exert a pronounced effect on preferences and evaluations. Conversely, when involvement is high, instrumental concerns such as price, performance, reliability, and risk reduction tend to dominate the evaluation process (Bettman, 1979). Evidence consistent with dual-process models suggests that identity-based effects diminish under conditions of high elaboration, high perceived risk, or substantial financial stakes (Petty & Cacioppo, 1986; Cleveland et al., 2015).

Related behavioral research also indicates that reliance on identity cues may decrease when cognitive load is high or when more deliberative cognitive systems are engaged (Forehand et al., 2011). Thus, product involvement and decision complexity can be understood as boundary conditions for identity-driven consumption: they help explain why consumers may readily express solidarity with minority-owned businesses in everyday, low-stakes contexts yet behave more pragmatically when stakes are higher or decisions are more consequential.

### 2.5. Integrative Review and Identification of the Research

An integrative examination of the existing literature indicates that minority entrepreneurship, ethnic identity, and consumer behavior have each generated substantial scholarly attention, yet these streams of research have seldom been brought together into a cohesive explanatory framework. On the supply side, studies have concentrated primarily on structural and managerial determinants of minority business performance, often conceptualizing minority status as a demographic risk factor rather than as a construct with meaningful psychological implications (Lussier, 1995; Gyimah et al., 2019). On the demand side, research on ethnic identity, consumer ethnocentrism, and identity-based consumption has demonstrated how group identification shapes preferences, evaluations, and loyalty. However, far less empirical work has examined how these identity-driven processes translate into sustained support for minority-owned businesses as entrepreneurial entities (Sharma et al., 1994; Cleveland et al., 2015).

In addition, evidence remains limited regarding the role of decision context and product involvement in conditioning identity-based motives. While theoretical perspectives suggest that identity effects should be more pronounced in low-involvement, symbolic, or routine consumption and less influential in high-involvement, risk-intensive decisions, relatively few studies have systematically investigated these dynamics in relation to minority-owned firms. Moreover, markets characterized by layered political, cultural, and social identities remain insufficiently explored, leaving open important questions about how identity-driven consumption unfolds in complex multicultural environments.

The present research seeks to address these gaps by examining how ethnic identification shapes consumer preferences, evaluations, and loyalty toward minority-owned businesses across varying levels of product involvement. By integrating insights from minority entrepreneurship, social identity theory, and consumer involvement frameworks, this work provides a more nuanced understanding of when and how identity-based motives translate into supportive marketplace behavior toward underrepresented entrepreneurs.

## 3. Theoretical Background and Hypotheses Development

### 3.1. Ethnic Identity and the Consumer Self-Concept

Ethnic identity represents a core component of self-definition that shapes how individuals think, feel, and behave as members of social groups (Phinney, 1992). Social identity theory proposes that individuals categorize themselves and others into social groups to preserve a positive self-concept and derive self-esteem from group membership (Tajfel & Turner, 1979). Accordingly, ethnicity functions as a salient marker of belonging and psychological meaning, shaping how individuals interpret social interactions and marketplace encounters (Crocker & Luhtanen, 1992).

In consumer behavior research, the self-concept has been established as a central driver of consumption choices. Consumers frequently select products and services that reflect or reinforce who they are and what they stand for (Belk, 1988; Aaker, 1999). Identity-based motivation theory further suggests that when a particular identity becomes salient, individuals are motivated to behave in ways that affirm it (Oyserman, 2009; Reed et al., 2012). In multicultural contexts, ethnic identity thus operates as a psychological filter that shapes perceptions of similarity, trust, and social alignment (Askegaard et al., 2005).

Within this framework, consumers who strongly identify with their ethnic group may view in-group businesses as extensions of themselves. These identity-consistent perceptions increase the likelihood of preferring, supporting, and defending minority-owned businesses (Light & Gold, 2000; Dana, 2011).

**H1.**  *Consumers with stronger ethnic identification will demonstrate a higher preference for minority-owned businesses compared to majority-owned businesses.*

### 3.2. Ethnic Identity, Store Evaluation, and Customer Loyalty

Ethnic identity also shapes evaluative and relational components of consumer judgment. According to the collective self-esteem perspective, individuals experience emotional benefit from positive associations with their group (Crocker & Luhtanen, 1992). When interacting with minority-owned businesses that reflect their cultural identity, consumers often perceive such firms as more trustworthy, fair, and socially responsible (Cleveland et al., 2015; Grier et al., 2019).

From a consumer psychology standpoint, identity-congruent evaluations occur through affective alignment: individuals evaluate in-group products more favorably because doing so reinforces their self-concept and reduces intergroup tension (Russell & Russell, 2006). Such processes extend to brand attachment, satisfaction, and long-term loyalty, forming deeper relational bonds between consumers and businesses (Stayman & Deshpandé, 1989; Berger & Heath, 2007).

Moreover, loyalty toward in-group entrepreneurs often carries a moral element. Supporting minority-owned enterprises can be construed as an act of empowerment and social reciprocity within the community (Bates, 2011). Studies of ethnic and minority markets demonstrate that cultural affinity can produce stable consumer–entrepreneur relationships that sustain local business ecosystems (Baycan-Levent & Nijkamp, 2009; Fairlie & Robb, 2008).

**H2.**  *Ethnic identification will be positively associated with favorable evaluations and greater loyalty toward minority-owned businesses.*

### 3.3. The Moderating Role of Product Involvement

Identity-based behavior depends not only on the strength of identification but also on the cognitive and emotional demands of the decision context. Product involvement refers to the perceived personal relevance, risk, and emotional investment associated with a purchase (Zaichkowsky, 1985; Laurent & Kapferer, 1985). Low-involvement decisions tend to rely on affective heuristics and symbolic cues rather than analytical processing (Petty et al., 1983; Celsi & Olson, 1988). In such contexts, identity cues, including ownership or cultural alignment, exert stronger influence on preferences and evaluations.

By contrast, high-involvement decisions emphasize functionality, reliability, risk reduction, and deliberation, reducing reliance on identity-based motives (Ahmed et al., 2004). This distinction aligns with dual-process models of persuasion, which suggest that identity effects weaken as cognitive elaboration increases (Petty & Cacioppo, 1986; Cleveland et al., 2015). Consequently, identity-driven support for minority-owned businesses is expected to be more pronounced in everyday, symbolic, or low-risk consumption contexts.

**H3.**  *The positive effect of ethnic identification on consumer preferences and evaluations will be stronger for low-involvement products than for high-involvement products.*

### 3.4. Conceptual Framework

Drawing on social identity theory (Tajfel & Turner, 1979), identity-based motivation theory (Oyserman, 2009), and consumer involvement models (Zaichkowsky, 1985; Laurent & Kapferer, 1985), this study proposes a conceptual framework explaining how ethnic identification shapes supportive consumer behavior toward minority-owned businesses.

The model posits that ethnic identity functions as a motivational driver that influences preferences, evaluations, and loyalty. Product involvement moderates this relationship by shaping when consumers rely on identity-based versus utilitarian considerations. These mechanisms together clarify how individual identity contributes to the social and economic sustainability of minority entrepreneurship. The framework also aligns with social capital perspectives, emphasizing shared identity and community-based trust as resources supporting entrepreneurial ecosystems (Putnam, 2000; Nahapiet & Ghoshal, 1998). Table 1 presents the conceptual definitions of the key constructs used in this research and the representative theoretical foundations on which they rely.

## 4. Study 1

### 4.1. Purpose

The purpose of this study is to examine the relationship between ethnic identity and consumer preferences for businesses perceived as minority-owned versus majority-owned. Specifically, the study tests whether individuals with stronger ethnic identification are more likely to prefer purchasing from minority-owned businesses. In addition, the study assesses whether this relationship varies across product types that differ in involvement level, comparing routine, low-involvement purchases (e.g., supermarkets, restaurants) with high-involvement purchases (e.g., computers, banking services). Demographic variables (gender, age, income) were also examined as potential covariates. The study was conducted in a Middle Eastern socio-cultural context marked by ethnic diversity and complex intergroup relations, which provides a meaningful setting for examining identity-based consumption.

### 4.2. Method

One hundred and seven respondents (48 men and 59 women), aged 17 to 50 (*M* = 30), participated in a telephone survey administered by a research company specializing in data collection within minority communities. All respondents completed the questionnaire in their native language.

Participants were presented with four purchasing scenarios designed to capture decisions across different involvement levels: buying products at a supermarket, reserving a restaurant for a family celebration, purchasing a personal computer, and opening a bank account. The supermarket and restaurant scenarios represented low-involvement consumption, whereas the computer and banking scenarios represented high-involvement consumption (Ahmed et al., 2004). For each scenario, respondents selected one preferred business from four options, two perceived as majority-owned and two perceived as minority-owned. These options were derived from a preliminary open-ended study conducted with twenty respondents from the same minority group, in which the most frequently mentioned businesses were selected for inclusion in the main survey. After choosing a preferred business, respondents indicated the primary reason for their decision from a predefined list that included price, product quality, convenience, product diversity, management style, trust, cultural compatibility, and other considerations.

Ethnic identity was measured using six items rated on a five-point Likert scale (1 = strongly disagree, 5 = strongly agree), including statements such as “I consider myself a member of my ethnic group,” “I feel very proud of my ethnic cultural background,” and “I am very attached to my ethnic culture.” The items were averaged to create an ethnic identity index (Cronbach’s α = 0.78), based on the measure developed by Laroche et al. (2007) and frequently applied in prior research (e.g., Kim et al., 2001; Josiassen, 2011; Shoham et al., 2017). Participants also reported demographic information, including gender, age, and monthly income level (1 = much lower than average, 7 = much higher than average).

In all scenarios, minority-owned businesses referred specifically to businesses owned by members of the same ethnic group as the respondents. This classification was based on widely shared community-level recognition of ownership rather than on a formal or legal definition of minority status and did not include businesses owned by other minority groups. In this study, the distinction between minority-owned and majority-owned businesses reflects how ownership is commonly perceived and socially recognized by consumers in the local marketplace. This operationalization is based on salient ownership cues and shared community understanding, rather than on formal legal ownership structures, shareholder composition, or workforce characteristics.

### 4.3. Results and Discussion

The findings indicated that the ethnic identity index was generally high among participants (*M* = 4.63, *SD* = 0.59; median = 5.0), with only 4.7% scoring below 4.0. No significant gender differences were observed in identity levels, and age did not predict ethnic identification (all *ps* > 0.10), suggesting that identification strength was relatively stable across demographic subgroups. Choice data were analyzed using binary logistic regression models, which are appropriate for modeling dichotomous preferences between minority-owned and majority-owned businesses across purchase scenarios.

In the supermarket scenario, 56% of respondents preferred majority-owned supermarkets and 44% preferred minority-owned ones. The most frequently cited reasons for their choices included price (25%), product quality (23%), and convenience (25%). Logistic regression analyses showed that none of the business-related predictors significantly explained the choice between store types. However, ethnic identity emerged as a significant predictor of preference for minority-owned supermarkets (*B* = 0.99, *SE* = 0.44, Wald = 4.96, *p* < 0.05), indicating that each point increase in ethnic identification was associated with a 2.68-fold increase in the odds of choosing a minority-owned business. Demographic variables (gender, age, income) did not moderate this effect.

In the restaurant scenario, 76% of respondents preferred minority-owned restaurants, and 24% preferred majority-owned ones. Choices were based primarily on food quality (31%), convenience (28%), and menu variety (22%), with price cited by only 4%. Ethnic identity did not significantly predict restaurant choice (*p* > 0.10). However, item-level analyses of the identity scale revealed that cultural attachment, specifically endorsing statements such as “My ethnic culture has the most positive impact on my life” and “I am very attached to my ethnic culture”, significantly predicted preference (*ps* < 0.05). These results are consistent with prior work showing that cultural connectedness influences support for in-group food establishments.

In the computer purchase scenario, 77% of respondents preferred minority-owned stores, while 23% preferred majority-owned ones. Product quality (31%), convenience (25%), price (19%), and product diversity (21%) were the most frequently cited considerations. Logistic regression showed that product quality was the only significant predictor of preference (*B* = 2.74, *SE* = 1.24, *p* < 0.05), with respondents emphasizing that quality was substantially more likely to choose a minority-owned store (Exp(B) = 15.50). Ethnic identity had no significant effect (*p* > 0.10), suggesting that high-involvement, performance-oriented decisions tend to attenuate identity-based influences.

A similar pattern emerged in the banking scenario. While 71% preferred majority-owned banks and 29% preferred minority-owned banks, choices were driven primarily by financial terms (40%) and convenience (32%). Logistic regression indicated that financial terms significantly predicted preference (*B* = 1.83, *SE* = 0.82, *p* < 0.05). Ethnic identity did not significantly influence bank choice (*p* > 0.10). Gender and age showed supplementary effects: men were more likely than women to prefer minority-owned banks (Exp(*B*) = 2.57), whereas older participants were less likely to do so.

Taken together, these findings demonstrate that ethnic identity significantly predicts supportive consumer choices in low-involvement contexts such as supermarkets, and that specific facets of cultural attachment predict restaurant preferences. In contrast, identity effects were absent in high-involvement contexts, where functional and financial considerations dominated. This pattern supports the theoretical expectation that identity-driven behavior is more likely to surface in routine, low-stakes decisions, whereas higher-stakes decisions activate more deliberative, utilitarian considerations, thereby reducing the influence of social identification on consumer choices.

### 4.4. Summary and Contribution of Study 1

Study 1 examined whether ethnic identity predicts preference for minority-owned versus majority-owned businesses and whether this pattern varies by purchase context. Respondents reported high levels of ethnic identification. Ethnic identity significantly predicted preference in low-involvement contexts, particularly supermarket purchases, and item-level cultural attachment predicted restaurant choice. In contrast, for high-involvement contexts such as computers and banking, practical considerations were more decisive and identity effects were not observed. Methodologically, the study uses real market categories and a validated identity index. Conceptually, it suggests that identity-based support for minority businesses is most evident in routine, culturally expressive consumption while attenuating in utilitarian, higher-stakes decisions.

## 5. Study 2

### 5.1. Purpose

The second study aimed to extend the empirical findings of Study 1 by testing whether the relationship between ethnic identity and consumer support for minority-owned businesses depends on the level of product involvement. Specifically, this study examined whether individuals with stronger ethnic identification demonstrate higher store evaluations and customer loyalty toward minority-owned enterprises, and whether this relationship differs between high- and low-involvement products.

### 5.2. Method

#### 5.2.1. Pretest: Validating Product Involvement

A preliminary online experiment was conducted through the MTurk platform to confirm that the two selected products, a refrigerator and a mobile phone charger, represent distinct levels of consumer involvement. The pretest included 156 participants (86 men and 70 women; average age 38), all of whom received financial compensation. Half of the participants were asked to assume they were considering the purchase of a refrigerator, and the other half were asked to assume they were considering the purchase of a mobile phone charger. Participants then indicated their agreement with ten statements regarding the purchase process (1 = strongly disagree, 7 = strongly agree), such as “I would be interested in reading about this product,” “I have compared product characteristics among brands,” and “I usually take many factors into account before purchasing this product.” These items, adapted from McQuarrie and Munson (1992), formed a reliable product involvement index (α = 0.89). The pretest results confirmed that the refrigerator was perceived as a high involvement product, whereas the mobile phone charger was perceived as a low-involvement product. The involvement index for the refrigerator condition (*M* = 5.28, *SD* = 0.81) was significantly higher than for the charger condition (*M* = 4.94, *SD* = 1.25), *t*(154) = 1.98, *p* < 0.05. The difference corresponds to a small to moderate effect size (Cohen’s d = 0.32). No gender differences were observed. These findings validated the selection of the two products as representing different involvement levels.

#### 5.2.2. Main Study: Participants and Procedure

The main study included 133 respondents from minority communities (40 men and 93 women), aged 21 to 65 (*M* = 35). The study was conducted in a Middle Eastern socio-cultural context characterized by ethnic diversity and salient group identities. Data were collected through a telephone survey administered by a research company specializing in minority populations. No significant effects of gender or income were detected for the key variables. Participants completed a structured questionnaire in their native language. Each respondent was randomly assigned to one of two hypothetical purchase scenarios. In the high involvement condition, participants imagined purchasing a refrigerator from a well-known minority-owned chain of electrical products. In the low-involvement condition, participants imagined purchasing a mobile phone charger from the same chain. The minority-owned retail chain described in the scenario was owned by members of the same ethnic group as the respondents and was commonly recognized within the community as such. The study therefore examined in-group support rather than generalized support for minority-owned businesses.

#### 5.2.3. Measures: Store Evaluation

Respondents rated eight items on a five-point scale (1 = strongly disagree, 5 = strongly agree) assessing perceptions of store management, customer orientation, innovativeness, and social responsibility. Representative items included “The store is well managed,” “The store provides excellent service,” and “The store contributes to the environment and community.” These items were averaged to form a store evaluation index (α = 0.90).

#### 5.2.4. Measures: Overall Store Assessment

Participants also evaluated the store on four seven-point items measuring overall evaluation (1 = very bad, 7 = excellent), product prices and costs, perceived value relative to price, and perceived fairness (1 = completely unfair, 7 = fair to a very large extent).

#### 5.2.5. Measures: Customer Loyalty

Loyalty was assessed using two five-point items: “I would recommend others to purchase products from the store” and “I would recommend others to increase their spending in this store.” These items were averaged to form a loyalty index (α = 0.83).

#### 5.2.6. Measures: Ethnic Identity

Two validated scales were used. The first was the Laroche ethnic identity index (Laroche et al., 2007), originally consisting of six items on a five-point agreement scale. One item, “I think of myself as belonging to my ethnic group first and to the larger society second”, was removed to improve reliability, yielding a five-item index (α = 0.89). The second measure was the Phinney ethnic identity index (Phinney, 1992; Al Ganideh & Awudu, 2021), consisting of four items capturing belonging, pride, and attachment to one’s ethnic group (α = 0.85).

#### 5.2.7. Demographics

Participants reported their age, gender, and income level (1 = much lower than average, 7 = much higher than average).

### 5.3. Results and Discussion

Overall, the level of ethnic identity was high. The Laroche index averaged 4.21 (*SD* = 0.71; median = 4.2), and the Phinney index averaged 4.09 (*SD* = 0.70; median = 4.0). Only 13.5% of participants scored below 4.0 on either index. No significant gender differences were observed for either measure (Laroche: *M* men = 4.22, *SD* = 0.86; *M* women = 4.21, *SD* = 0.64, *t*(131) < 1, *p* > 0.10; Phinney: *M* men = 4.18, *SD* = 0.80; *M* women = 4.05, *SD* = 0.65, *t*(131) = 1.03, *p* > 0.10). Age did not significantly predict ethnic identity (Laroche: *F* = 1.79, *p* > 0.10; Phinney: *F* < 1, *p* > 0.10).

A *t* test comparing the two experimental conditions revealed significant differences in ethnic identity: respondents in the refrigerator condition reported higher ethnic identity (Laroche *M* = 4.51, *SD* = 0.57; Phinney *M* = 4.29, *SD* = 0.63) than those in the mobile phone charger condition (Laroche *M* = 3.95, *SD* = 0.72; Phinney *M* = 3.90, *SD* = 0.70), *t*(131) = 4.95, *p* < 0.001, and *t*(131) = 3.32, *p* < 0.001, respectively. Participants in the refrigerator condition were also older (*M* = 37.65, *SD* = 9.90) than those in the charger condition (*M* = 32.90, *SD* = 8.04), *t*(131) = 3.05, *p* < 0.01. These differences indicate that the experimental conditions were not fully equivalent in terms of age and baseline ethnic identity.

No significant differences were found between product conditions for store evaluation, overall store rating, prices and costs, customer value, fairness, or loyalty (all *p* > 0.10).

Six linear regressions were conducted for each dependent variable, store evaluation, overall rating, price and cost evaluation, perceived value, fairness, and loyalty, using product type (dummy coded: 1 = refrigerator, 0 = charger), Laroche ethnic identity index, and their interaction as predictors. As shown in Table 2, product type and the interaction were non-significant in all models. Ethnic identity, however, was positively associated with store evaluation and loyalty. The association approached significance for price and cost evaluation (*p* = 0.08) and was significant (*p* < 0.05) for the other five models. It is also possible that the relatively modest separation between the involvement conditions limited the activation of distinct psychological processes, which may help explain the absence of moderation effects in Study 2.

A parallel set of analyses using the Phinney ethnic identity index as the predictor produced similar results (Table 3).

Additional twelve regressions were conducted to test the moderate effect of age by including it and its interactions with product type and ethnic identity. As shown in Table 4 and Table 5, age did not significantly affect most outcomes, except for product price and cost evaluations, where older participants reported more favorable assessments (*p* < 0.05).

Together, these results indicate that higher ethnic identity levels were associated with more positive evaluations and stronger loyalty toward minority-owned businesses, independent of product involvement level. Product type and its interaction with ethnic identity were non-significant, suggesting that identity-based consumer support extends beyond low-involvement contexts.

## 6. Discussion

The present research examined how ethnic identification shapes consumer support for minority-owned businesses within culturally diverse markets. Across two empirical studies, the findings offer convergent evidence that ethnic identity meaningfully influences consumption patterns and evaluations of minority-owned enterprises. Importantly, the results indicate that identity-driven consumer behavior is not uniform across all product categories; rather, it varies according to the symbolic meaning and perceived involvement of the purchase context. The studies were conducted in a Middle Eastern socio-cultural context characterized by ethnic diversity and salient group identities, which provides a meaningful setting for examining identity-based supportive consumption. This context is likely to heighten the salience of ethnic identification in everyday marketplace interactions and thus informs the interpretation of the present findings.

Study 1 demonstrated that individuals with stronger ethnic identification were more likely to prefer minority-owned businesses in low-involvement, routine purchase situations such as grocery shopping and restaurant selection. These findings suggest that ethnic identity is expressed more strongly when consumption is symbolic, habitual, or socially expressive, rather than when decisions are economically consequential. Study 2 extended these insights by showing that ethnic identification was associated with higher store evaluations and greater customer loyalty toward minority ventures or ethnic enterprises operating within their communities. Although product involvement did not significantly moderate these relationships in Study 2, the consistent positive effects of ethnic identity across evaluative and attitudinal measures reinforce the proposition that identity salience translates into supportive marketplace behavior. It is important to note that support for minority-owned businesses is operationalized differently across the two studies. Whereas Study 1 captures behavioral support through choice in simulated purchase scenarios, Study 2 focuses on evaluative and intentional forms of support, including store evaluations and loyalty intentions.

It is also possible that, particularly in food-related contexts, minority ownership functions as a cue of perceived authenticity, which may reinforce consumer preferences alongside ethnic identification. In such cases, authenticity perceptions may shape expectations regarding cultural fit, quality, or experiential value. Although authenticity was not directly measured in the present studies, future research could examine how identity-based processes and perceived authenticity jointly influence supportive consumption toward minority-owned businesses.

Theoretically, this research advances the literature by integrating two domains that are rarely examined together: minority entrepreneurship and consumer identity. Whereas previous entrepreneurship frameworks emphasized financial, managerial, and structural factors in determining business performance (Lussier, 1995; Halabi & Lussier, 2014; Hyder & Lussier, 2016), the current studies highlight that socio-psychological factors, particularly ethnic identity, also shape market outcomes in meaningful ways. From a consumer behavior perspective, the results extend work on self-concept and social identity processes (Reed, 2002; Berger & Heath, 2007) by illustrating that ethnic identification can act as a form of social capital that benefits minority entrepreneurs through enhanced trust, perceived similarity, and community-based solidarity.

More broadly, the observed pattern of alignment between ethnic identification, evaluative judgments, and supportive behavioral intentions is consistent with principles of cognitive consistency, whereby individuals strive for coherence between their self-concept and marketplace responses. While the present research focuses specifically on ethnic identity, similar identity-based processes may operate for other salient social identities, such as gender, national origin, or social class, when these identities are psychologically central to the self. Importantly, the current studies do not test these extensions empirically. Rather, they suggest that identity-consistent consumer behavior may reflect a more general mechanism of self-concept alignment that warrants examination across different social identity domains in future research.

The findings also clarify the contextual nature of identity-based consumption. Research on product involvement (Laurent & Kapferer, 1985; Celsi & Olson, 1988) suggests that high-involvement decisions typically activate analytical, risk-sensitive evaluations, thereby reducing reliance on social or identity cues. Conversely, low-involvement consumption permits identity-relevant motives, affective, heuristic, or habitual, to guide decisions (Belk, 1988; Cleveland et al., 2015). The patterns observed here help reconcile mixed findings in the literature by showing that identity effects are strongest in everyday, low-stakes contexts and less influential in high-stakes, utilitarian decisions.

This research further refines the conceptualization of supportive consumption by demonstrating that it manifests both behaviorally and attitudinally. Previous studies often treated identity as a demographic characteristic (Aldrich & Waldinger, 1990; Dana, 2011), but the present research positions ethnic identity as an active psychological mechanism influencing evaluations, preferences, and loyalty toward minority-owned enterprises. This insight expands traditional entrepreneurship models that focus narrowly on economic predictors, suggesting that identity dynamics should be incorporated more explicitly into theories of entrepreneurial success among minority populations.

Several limitations warrant consideration. First, the studies were conducted within a specific socio-cultural environment, potentially limiting the generalizability of the findings to other minority groups or markets. Second, both studies relied on self-report measures, which may be influenced by social desirability or perceived social norms. Future research should incorporate behavioral data or longitudinal designs to capture actual purchasing patterns over time. Additional moderators, such as perceived discrimination, intergroup contact, or the relative salience of national versus ethnic identity, may also help illuminate boundary conditions of identity-based consumer support.

Overall, the findings provide a theoretically grounded and empirically supported framework for understanding how ethnic identity drives consumer support for minority entrepreneurs. The research demonstrates that marketplace inclusion is shaped not only by structural opportunity but also by shared identity, community belonging, and collective meaning. The following section discusses the practical and policy implications that arise from these findings.

## 7. Conclusions and Implications

### 7.1. Conclusions

This research advances current understanding of how ethnic identity shapes consumer support for minority-owned businesses within culturally diverse market environments. Across two empirical studies, the findings demonstrate that identity-driven consumption operates as a psychological and social mechanism through which collective belonging facilitates economic participation. Consumers reporting stronger ethnic identification exhibited greater supportive tendencies toward minority entrepreneurs, particularly in consumption contexts that are more expressive, habitual, or symbolic in nature.

By integrating insights from social identity theory, consumer behavior, and entrepreneurship studies, this research provides a more complete theoretical account of inclusion in marketplace dynamics. It reveals that ethnic identity is not merely a descriptive demographic marker, but a meaningful socio-psychological resource capable of influencing evaluative judgments, loyalty, and preferences. As such, the studies underscore the role of identity in sustaining community-based entrepreneurship and promoting equitable participation in local economic ecosystems.

### 7.2. Practical and Theoretical Implications

From a theoretical perspective, the results reinforce the view that ethnic identity operates as a socio-cognitive mechanism shaping consumer decision making. While traditional entrepreneurship frameworks have emphasized financial, managerial, or structural determinants of success (Lussier, 1995; Halabi & Lussier, 2014), the present findings show that cultural and identity-based processes also influence outcomes in meaningful ways. This supports conceptualizations of entrepreneurship as socially embedded, where community ties, cultural meaning, and shared identities interact with economic factors to shape venture sustainability (Aldrich & Waldinger, 1990).

Furthermore, the integration of identity theory with product involvement offers a more nuanced lens for understanding when identity-based consumption emerges. Consistent with consumer research (Celsi & Olson, 1988; Laurent & Kapferer, 1985), the findings suggest that identity salience is more influential in low-involvement contexts, where decisions rely on affective or habitual heuristics. This provides an important bridge between psychological models of self-concept (Reed, 2002) and economic models of consumer choice, highlighting the contextual nature of supportive consumption.

From a practical standpoint, the findings offer several meaningful implications for minority entrepreneurs. Businesses operating within culturally diverse markets may benefit from emphasizing cultural authenticity, shared identity markers and sustained community engagement, as these elements can strengthen consumer trust and foster long-term loyalty. The use of symbolic signals, including language, cultural design, and community narratives, may further enhance the perceived legitimacy of minority-owned ventures among consumers who share the same ethnic background. In addition, developing branding strategies that align with collective identity can assist minority-owned firms in differentiating themselves within competitive market environments.

The findings of this research also carry important implications for policymakers. Initiatives intended to support minority entrepreneurship should extend beyond conventional financial or technical assistance and include efforts aimed at improving visibility, promoting representation and facilitating deeper integration within community networks and public marketplaces. Recognizing ethnic identity as a social and psychological resource, rather than merely a demographic attribute, can enable the design of more inclusive and sustainable approaches to economic development. Furthermore, policies that encourage cross-cultural engagement may strengthen consumer empowerment while simultaneously contributing to the long-term viability of minority-owned businesses within multicultural societies.

## 8. Limitations and Future Research

Despite its contributions, this research has several limitations that open pathways for future inquiry. First, both studies were conducted within a single socio-cultural context, which may limit the generalizability of the findings to other minority populations or multicultural environments. In addition, both studies relied on relatively modest sample sizes and survey-based sampling through a research company, which may limit representativeness and the generalizability of the findings. Future research could replicate the studies across diverse ethnic groups and national contexts to assess contextual boundary conditions and further examine how identity-driven consumption unfolds across different market settings. In addition, the present research focuses on subjective ethnic identification and does not differentiate between ethnic identity and immigration-related factors such as immigrant status, generational position, or level of acculturation. Although these dimensions are often related, they are conceptually distinct and may shape consumer perceptions and marketplace behavior in different ways. Future research would benefit from disentangling ethnic identification from immigration-related characteristics in order to examine how these factors jointly or independently influence supportive consumption toward minority-owned businesses.

Second, the present studies relied on self-reported measures of preference, evaluation, and loyalty. Although self-reports are standard in consumer research, they may be influenced by social desirability, implicit bias, identity priming, or respondents’ desire to present themselves positively. Future studies could therefore employ behavioral designs, including incentive-compatible choice tasks, field experiments in real retail settings, or digital trace data capturing actual purchasing patterns over time. Longitudinal designs would further enable examination of how supportive consumption emerges, stabilizes, or declines in response to identity salience, market events, or shifts in socio-political climate.

Third, the current research conceptualizes ethnic identity as the central psychological driver of supportive behavior. However, identity is multifaceted, dynamic, and often intertwined with other social constructs. Future research could examine how perceived discrimination, minority stress, or intergroup threat heighten or suppress identity-based consumption. Additionally, constructs such as bicultural identity integration, collective efficacy, acculturation orientation, or political identity may moderate the strength of identity-driven preferences, offering a richer psychological framework for understanding supportive consumption.

Finally, the present research focused primarily on consumers, while identities also shape the behavior and strategic choices of entrepreneurs. Future research could adopt a dual-perspective or relational identity approach, examining how minority business owners engage with identity-related cues to build trust and legitimacy in the marketplace. Investigating identity processes on both sides of the exchange may offer a more holistic understanding of how identity operates in market interactions and contributes to the sustainability of minority-owned enterprises.

## Figures and Tables

**Table 1:** Conceptual Definitions and Theoretical Foundations of Key Constructs.

Construct	Conceptual Definition	Representative References
Ethnic Identity	A component of self-concept derived from an individual’s sense of belonging, attachment, and pride in their ethnic group, which shapes perceptions of similarity, trust, and in-group behavior in the marketplace.	Phinney (1992); Tajfel and Turner (1979); Oyserman (2009); Reed et al. (2012); Askegaard et al. (2005)
Product Involvement	The perceived personal relevance, importance, and risk associated with a product or purchase decision, determining the degree of cognitive and emotional engagement in the consumption process.	Zaichkowsky (1985); Laurent and Kapferer (1985); Petty et al. (1983)
Supportive Consumption toward Minority Entrepreneurs	Consumers’ attitudinal and behavioral tendency to prefer, evaluate, and remain loyal to businesses owned by members of their ethnic group, reflecting identity-congruent marketplace support.	Light and Gold (2000); Baycan-Levent and Nijkamp (2009); Bates (2011); Fairlie and Robb (2008); Grier et al. (2019)

**Table 2:** Regression results predicting store evaluation and customer loyalty using the Laroche ethnic identity index.

Dependent Variable	Independent Variables	B	S.E	Beta	R^2^
Store Reputation	product	0.17	0.84	0.12	0.09 **
	Laroche ethnic identity index	0.34 *	0.12	0.34 *	
	Product * Laroche ethnic identity index	−0.11	0.19	−0.34	
Overall Rating of the store	product	−2.13	1.85	−0.66	0.12 **
	Laroche ethnic identity index	0.71 **	0.25	0.31 **	
	Product * Laroche ethnic identity index	0.33	0.43	0.46	
Store’s Products Prices and Costs	product	−1.39	1.45	−0.57	0.08 *
	Laroche ethnic identity index	0.35	0.2	0.2	
	Product * Laroche ethnic identity index	0.32	0.33	0.61	
Customer’s Value	product	0.99	1.42	0.4	0.13 ***
	Laroche ethnic identity index	0.75 ***	0.2	0.43 ***	
	Product * Laroche ethnic identity index	−0.25	0.33	−0.47	
Fairness of the Store	product	0.93	1.54	0.36	0.08 *
	Laroche ethnic identity index	0.64 *	0.21	0.35 *	
	Product * Laroche ethnic identity index	−0.27	0.35	−0.48	
Customer Loyalty	product	−0.28	0.98	−0.17	0.10 **
	Laroche ethnic identity index	0.37 **	0.13	0.32 **	
	Product * Laroche ethnic identity index	0.04	0.22	0.11	

Note: * *p* < 0.05, ** *p* < 0.01, *** *p* < 0.0001.

**Table 3:** Regression results predicting store evaluation and customer loyalty using the Phinney ethnic identity index.

Dependent Variable	Independent Variables	B	S.E	Beta	R^2^
Store Reputation	product	0.44	0.74	0.31	0.12 **
	Phinney ethnic identity index	0.42 ***	0.12	0.41 ***	
	Product * Phinney ethnic identity index	−0.17	0.18	−0.52	
Overall Rating of the store	product	−1.80	1.65	−0.56	0.15 ***
	Phinney ethnic identity index	0.79 **	0.26	0.34 **	
	Product * Phinney ethnic identity index	0.29	0.4	0.39	
Store’s Products Prices and Costs	product	−0.77	1.31	−0.32	0.09 **
	Phinney ethnic identity index	0.41 *	0.2	0.23 *	
	Product * Phinney ethnic identity index	0.21	0.31	0.37	
Customer’s Value	product	1.48	1.28	0.6	0.15 ***
	Phinney ethnic identity index	0.81 ***	0.2	0.46 ***	
	Product * Phinney ethnic identity index	−0.36	0.31	−0.63	
Fairness of the Store	product	1.02	1.39	0.39	0.10 **
	Phinney ethnic identity index	0.71 **	0.22	0.38 **	
	Product * Phinney ethnic identity index	−0.29	0.33	−0.49 **	
Customer Loyalty	product	0.26	0.89	0.15	0.10 **
	Phinney ethnic identity index	0.41 **	0.14	0.34 **	
	Product * Phinney ethnic identity index	−0.07	0.21	−0.19	

Note: * *p* < 0.05, ** *p* < 0.01, *** *p* < 0.0001.

**Table 4:** Regression results including age as moderator (Laroche ethnic identity index).

Dependent Variable	Independent Variables	B	S.E	Beta	R^2^
Store Reputation	product	−0.34	2.94	−0.24	0.09
	Laroche ethnic identity index	0.55	0.43	0.55	
	Age	0.02	0.05	0.3	
	Product * Laroche ethnic identity index	−1.00	0.68	−0.32	
	Product * Age	0.01	0.08	0.25	
	Laroche ethnic identity index * Age	−0.01	0.01	−0.47	
	Product * Laroche ethnic identity index * Age	0	0.02	0.14	
Overall Rating of the store	product	−3.70	6.53	−1.16	0.13 *
	Laroche ethnic identity index	0.67	0.96	0.29	
	Age	0	0.12	0.02	
	Product * Laroche ethnic identity index	0.83	1.5	1.18	
	Product * Age	0.04	0.17	0.47	
	Laroche ethnic identity index * Age	0	0.03	0.04	
	Product * Laroche ethnic identity index * Age	−0.01	0.04	−0.73	
Store’s Products Prices and Costs	product	6.61	4.97	2.7	0.13 *
	Laroche ethnic identity index	2.10 **	0.73	1.22 **	
	Age	0.23 *	0.09	1.77 *	
	Product * Laroche ethnic identity index	−1.48	1.14	−2.77	
	Product * Age	−0.25	0.13	−4.04	
	Laroche ethnic identity index * Age	−0.06 *	0.02	−2.21 *	
	Product * Laroche ethnic identity index * Age	0.06	0.03	4.21	
Customer’s Value	product	0.14	4.99	0.06	0.14 **
	Laroche ethnic identity index	1.32	0.74	0.75	
	Age	0.07	0.09	0.54	
	Product * Laroche ethnic identity index	−0.13	1.15	−0.24	
	Product * Age	0.01	0.13	0.11	
	Laroche ethnic identity index * Age	−0.02	0.02	−0.71	
	Product * Laroche ethnic identity index * Age	0	0.03	0.04	
Fairness of the Store	product	−1.14	5.43	−0.44	0.09
	Laroche ethnic identity index	0.99	0.8	0.54	
	Age	0.04	0.1	0.3	
	Product * Laroche ethnic identity index	0.04	1.25	0.07	
	Product * Age	0.05	0.14	0.69	
	Laroche ethnic identity index * Age	−0.01	0.02	−0.41	
	Product * Laroche ethnic identity index * Age	−0.01	0.03	−0.42	
Customer Loyalty	product	−0.81	3.45	−0.49	0.1
	Laroche ethnic identity index	0.32	0.51	0.27	
	Age	−0.01	0.06	−0.07	
	Product * Laroche ethnic identity index	0.12	0.79	0.32	
	Product * Age	0.02	0.09	0.37	
	Laroche ethnic identity index * Age	0	0.02	0.1	
	Product * Laroche ethnic identity index * Age	−0.00	0.02	−0.27	

Note: * *p* < 0.05, ** *p* < 0.01.

**Table 5:** Regression results including age as moderator (Phinney ethnic identity index).

Dependent Variable	Independent Variables	B	S.E	Beta	R^2^
Store Reputation	product	0.72	2.53	0.51	0.13 **
	Phinney ethnic identity index	0.61	0.42	0.6	
	Age	0.02	0.05	0.26	
	Product * Phinney ethnic identity index	−0.36	0.6	−1.10	
	Product * Age	−0.01	0.07	−0.37	
	Phinney ethnic identity index * Age	−0.01	0.01	−0.37	
	Product * Phinney ethnic identity index * Age	0.01	0.02	0.78	
Overall Rating of the store	product	−3.55	5.65	−1.11	0.16 **
	Phinney ethnic identity index	0.8	0.93	0.34	
	Age	0.01	0.11	0.07	
	Product * Phinney ethnic identity index	0.76	1.34	1.04	
	Product * Age	0.04	0.15	0.53	
	Phinney ethnic identity index * Age	0	0.03	0	
	Product * Phinney ethnic identity index * Age	−0.01	0.04	−0.65	
Store’s Products Prices and Costs	product	4.87	4.39	1.99	0.12 *
	Phinney ethnic identity index	1.81 *	0.72	1.03 *	
	Age	0.19 *	0.09	1.40 *	
	Product * Phinney ethnic identity index	−1.10	1.04	−1.97	
	Product * Age	−0.18	0.12	−2.87	
	Phinney ethnic identity index * Age	−0.04 *	0.02	−1.58 *	
	Product * Phinney ethnic identity index * Age	0.04	0.03	2.85	
Customer’s Value	product	3.78	4.36	1.53	0.16 **
	Phinney ethnic identity index	1.57 *	0.71	0.88 *	
	Age	0.1	0.09	0.71	
	Product * Phinney ethnic identity index	−0.99	1.04	−1.75	
	Product * Age	−0.08	0.12	−1.23	
	Phinney ethnic identity index * Age	−0.02	0.02	−0.84	
	Product * Phinney ethnic identity index * Age	0.02	0.03	1.43	
Fairness of the Store	product	0.49	4.71	0.19	0.12 *
	Phinney ethnic identity index	1.36	0.77	0.73	
	Age	0.08	0.09	0.57	
	Product * Phinney ethnic identity index	−0.35	1.12	−0.59	
	Product * Age	−0.00	0.13	−0.05	
	Phinney ethnic identity index * Age	−0.02	0.02	−0.69	
	Product * Phinney ethnic identity index * Age	0.01	0.03	0.39	
Customer Loyalty	product	1.94	3.02	1.16	0.11 *
	Phinney ethnic identity index	0.55	0.5	0.46	
	Age	0.02	0.06	0.22	
	Product * Phinney ethnic identity index	−0.56	0.72	−1.46	
	Product * Age	−0.05	0.08	−1.16	
	Phinney ethnic identity index * Age	−0.00	0.02	−0.24	
	Product * Phinney ethnic identity index * Age	0.01	0.02	1.43	

Note: * *p* < 0.05, ** *p* < 0.01.

## Data Availability

Data are available from the authors upon reasonable request.
